# Acquisition of Complement Inhibitor Serine Protease Factor I and Its Cofactors C4b-Binding Protein and Factor H by *Prevotella intermedia*

**DOI:** 10.1371/journal.pone.0034852

**Published:** 2012-04-13

**Authors:** Sven Malm, Monika Jusko, Sigrun Eick, Jan Potempa, Kristian Riesbeck, Anna M. Blom

**Affiliations:** 1 Section of Medical Protein Chemistry, Department of Laboratory Medicine, Skåne University Hospital, Lund University, Malmö, Sweden; 2 Laboratory of Oral Microbiology, Department of Periodontology, University of Bern, Bern, Switzerland; 3 Faculty of Biochemistry, Biophysics and Biotechnology, Department of Microbiology, Jagiellonian University, Krakow, Poland; 4 Center of Oral Health and Systemic Diseases, University of Louisville Dental School, Louisville, Kentucky, United States of America; 5 Medical Microbiology, Department of Laboratory Medicine, Skåne University Hospital, Lund University, Malmö, Sweden; Oxford University, United Kingdom

## Abstract

Infection with the Gram-negative pathogen *Prevotella intermedia* gives rise to periodontitis and a growing number of studies implies an association of *P. intermedia* with rheumatoid arthritis. The serine protease Factor I (FI) is the central inhibitor of complement degrading complement components C3b and C4b in the presence of cofactors such as C4b-binding protein (C4BP) and Factor H (FH). Yet, the significance of complement inhibitor acquisition in *P. intermedia* infection and FI binding by Gram-negative pathogens has not been addressed. Here we show that *P. intermedia* isolates bound purified FI as well as FI directly from heat-inactivated human serum. FI bound to bacteria retained its serine protease activity as shown in degradation experiments with ^125^I-labeled C4b. Since FI requires cofactors for its activity we also investigated the binding of purified cofactors C4BP and FH and found acquisition of both proteins, which retained their activity in FI mediated degradation of C3b and C4b. We propose that FI binding by *P. intermedia* represents a new mechanism contributing to complement evasion by a Gram-negative bacterial pathogen associated with chronic diseases.

## Introduction

*Prevotella intermedia* is regarded as an important causative agent of periodontitis besides *Aggregatibacter actinomycetemcomitans*, *Porphyromonas gingivalis*, *Tannerella forsythia* and *Fusobacterium nucleatum* [1]. Gingivitis and periodontitis are inflammatory diseases affecting the surrounding and supporting tissues of the teeth and caused by infection with microbial periodontal pathogens. Gingivitis is highly prevalent affecting up to 90% of the world's population and represents an inflammation limited to soft gingival tissues while periodontitis describes a severe manifestation of periodontal disease characterized by the formation of tissue pockets associated with irreversible damage of tissue and supporting bone, eventually leading to tooth loss [2]. Recent findings support the correlation between periodontitis and cardiovascular disease [3] and periodontal pathogens have been detected in the coronary arteries from patients afflicted with chronic periodontitis [4]. Moreover, clinical studies as well as the presence of DNA of periodontal pathogens in synovial fluid and serum, most frequently from *P. intermedia* and *P. gingivalis*, suggest a link between periodontitis and rheumatoid arthritis [5], [6].

The complement system is composed of a set of proteolytic enzymes and represents an essential part of innate immunity. It exhibits its bactericidal properties upon cascade-like activation reactions, which take place either through the classical pathway, the lectin pathway or the alternative pathway. Antibodies attached to pathogens or C1q bound to bacterial surfaces mediate complement activation through the classical pathway, whereas the lectin pathway is triggered by carbohydrate ligands. The alternative pathway represents an activation loop of the aforementioned pathways but can also be driven by the spontaneous tick-over of C3 to hydrolyzed C3(H_2_O) or properdin binding. Complement as a broad first line defense mechanism confronts pathogens with an effective bactericidal environment. Activation of complement leads either to opsonization of the pathogen for phagocytosis, lysis through the formation of the membrane attack complex, or the release of proinflammatory anaphylatoxins [7]. The omnipresent broad cytotoxicity of complement underlines the importance of controlling its activation by inhibitors in order to prevent self-damage to host tissues. Inhibitors such as C4b-binding protein (C4BP) and factor H (FH) inhibit the classical/lectin or the alternative pathway, respectively, by serving as cofactors in degradation of C4b and C3b by factor I (FI) as well as hindering the formation and accelerating the decay of the C3 convertases [8], [9]. The serine protease FI has a serum concentration of 35 mg/L and inhibits all complement pathways [10]. Its activity requires the presence of cofactors such as C4BP and FH. C4BP is found in human plasma at concentrations of ∼200 mg/L [11] while the concentration of FH in human plasma varies from 116 to 711 mg/L [11], [12]. Furthermore, serum proteins including complement are present in the gingival fluid from inflamed periodontal tissue [13] thus constituting partially also the proteins present in saliva [14].

Acquisition of complement inhibitors by various bacterial pathogens, mainly the soluble inhibitors C4BP and FH, leading to protection from activated complement, has been reported previously [15], [16]. Only a few periodontal pathogens have been reported to bind these cofactors; binding of FH occurs to preparations of the outer membrane protein 100 of *Aggregatibacter actinomycetemcomitans* [17], *P. gingivalis* binds C4BP [18] and *Treponema denticola* binds FH, which is subsequently cleaved by the protease dentilisin [19]. So far only *Staphylococcus aureus* has been shown to bind FI via clumping factor A (ClfA), which appears to act as a cofactor to FI in degradation of C3b [20], [21]. This leads to decreased phagocytosis efficiency by human polymorphonuclear cells [22].

Here we show for the first time binding of FI to a Gram-negative bacterial pathogen, *P. intermedia*. We also found that *P. intermedia* captures C4BP and FH. All three inhibitors retain their activity when bound to *P. intermedia* ATCC 25611. Thus, this study provides insight into a new evasion strategy of a main periodontal pathogen able to establish chronic infection.

## Materials and Methods

### Ethics statement

Normal human serum (NHS) was prepared from blood of healthy volunteers after written informed consent had been obtained with the specific permit by the ethics committee of Lund University (permit number 418/2008).

### Strains and culture conditions

*P. intermedia* OMZ 248 and OMZ 324, kindly provided by Ellen V. G. Frandsen, Department of Oral Biology, Royal Dental College, Aarhus, Denmark [23], MH6, isolated in Jena from a patient suffering from severe chronic periodontitis, as well as the type strain ATCC 25611 (American Type Culture Collection, Manassas, VA) were cultured on Fastidious Anaerobe agar plates for 4 days at 35°C in an anaerobic chamber (80% N_2_, 10% CO_2_, 10% H_2_) or in anaerobic jars containing an atmosphere depleted of oxygen using Anaerogen sachets (Oxoid, Basingstoke, UK). Several strains were employed as controls, with their culture conditions described below. *Streptococcus pyogenes* CCUG 25571, *Moraxella catarrhalis* RH4, *M. catarrhalis* Δ*usp*A1Δ*usp*A2 [24] were cultivated on blood agar plates (supplemented with zeocin and chloramphenicol for the mutant strain) at 37°C in an atmosphere enriched with 5% CO_2_. *S. aureus* ATCC 25923 as well as *S. aureus* Newman (wild type laboratory strain (T.J. Foster) were cultured on tryptic soy broth agar plates, and *Escherichia coli* DH5α was cultured on LB agar plates, all at 37°C in normal atmosphere. *Porphyromonas gingivalis* W50 [25] and W83 [26] were cultivated for 7–8 days at 35°C on FAA agar plates in an anaerobic chamber. During the initial screening of bacterial strains for their ability to recruit FI on their surface, *E. coli* DH5α exhibited only weak FI binding capacity, hence being chosen as an internal low binding control for each binding experiment. *S. aureus* ATCC 25923 is a strain associated with ClfA expression [27]–[29] and *S. aureus* Newman has been shown to bind FI [20], thus FI acquisition by these strains was anticipated. *Porphyromonas gingivalis* W50 and W83 [18] as well as *M. catarrhalis* RH4 bind C4BP [24], while *S. pyogenes* strains have been shown to capture FH on their surface [30]. Conversely, *S. aureus* ATCC 25923 has been reported not to bind FH [31]. Therefore, *S. aureus* ATCC 25923 and Newman (FI binding positive controls) as well as *E. coli* DH5α (FI binding negative control) served as control strains in FI binding experiments, *M. catarrhalis* RH4, *P. gingivalis* W50 and W80 (C4BP binding positive controls) and *M. catarrhalis* RH4 Δ*uspA*1/2 as well as *E. coli* DH5α (C4BP binding negative controls) were chosen to assess the binding capacity of *P. intermedia* strains in C4BP binding experiments. Likewise, *S. pyogenes* CCUG 25571 (FH binding positive control) and *S. aureus* ATCC 25923 and *E. coli* DH5α (FH binding negative controls) appeared to be appropriate internal controls investigating FH acquisition by *P. intermedia*.

### Proteins and antibodies

FI, C4BP and FH were purified from human serum as described previously [11], [32], [33]. When higher concentrations of FI were required for competition experiments recombinant FI expressed in eukaryotic cells was used [34]. C3met and C4met were derived from C3 and C4 respectively (Complement Technology, Tyler, Tx). Briefly, C3 and C4 were incubated with 0.1 M methylamine hydrochloride pH 8.0 for 1 h at 37°C and subsequently dialyzed against 50 mM Tris-HCl, 150 mM NaCl, pH 8.0. The polyclonal rabbit antibody PK9205 against human FI was raised in-house. Goat anti human FI antiserum A313 was purchased from Quidel (San Diego, CA) and rabbit anti goat horseradish peroxidase (HRP)-conjugated antibody was obtained from Dako (Glostrup, Denmark).

### Direct binding of ^125^I-labeled FI, C4BP and FH by *P. intermedia*

Serum purified proteins were labeled with ^125^I using Iodobeads (Pierce, Rockford, IL) or the chloramine T method [35] (specific activities between 0.22–0.35 MBq/µg protein). Binding experiments were essentially performed as described earlier [36]. Briefly, all *P. intermedia* as well as the control strains were harvested from plates and resuspended in phosphate buffered saline (PBS) (137 mM NaCl, 2.7 mM KCl, 1.47 mM KH_2_PO_4_, 8 mM Na_2_HPO_4_) supplemented with 1% bovine serum albumin (BSA), pH 7.0, to obtain an OD_600_ of 0.5. Bacteria were harvested and resuspended in 1/100 volume PBS with 1% BSA before proceeding with the binding assay. Twenty µl of the bacterial suspension (approximately 2×10^9^ bacteria) were mixed with 250 kcpm ^125^I-FI, or 500 kcpm ^125^I-C4BP and ^125^I-FH, respectively, and incubated for 1 h at RT in a total reaction volume of 40 µl. Protein bound to bacteria was separated from unbound protein by centrifugation through 250 µl of 20% sucrose for 3 min at 10,000 rpm (Biofuge 13, Heraeus Sepatech, Osterode, Germany). The radioactivity associated with pellets and supernatants was measured in a gamma counter (Gamma Master 1277, LKB Wallac, Turku, Finland). Samples containing ^125^I-labeled proteins that were mixed with buffer alone without bacteria served as negative controls.

In order to elucidate the specificity and to further characterize protein binding by *P. intermedia*, competition experiments with the type strain *P. intermedia* ATCC 25611 were performed. First, 20 µl bacterial suspension were mixed with unlabelled FI (final concentrations 0–5000 nM/0–0.44 µg/µl) as well as 250 kcpm ^125^I-FI in a total reaction volume of 40 µl and samples were incubated for 1 h at RT. Unbound protein was separated from the bacteria containing fraction by centrifugation through 20% sucrose and radioactivity measured as described above. In competition experiments with FH and C4BP likewise to the competition experiments with FI, 20 µl of the bacterial suspensions were mixed with labeled and unlabelled proteins in a total reaction volume of 40 µl. Binding of 500 kcpm ^125^I-FH was investigated in the presence of 7.5 µg (final concentration 1.25 µM) unlabeled FH, whereas binding of 250 kcpm ^125^I-C4BP was determined in the presence of 30 µg (final concentration 1.32 µM) unlabeled C4BP and bacterial suspensions that had been adjusted to an OD_600_ of 0.5 before. Otherwise, samples were treated as described for FI competition experiments. The sensitivity of the interaction to ionic strength was tested adding increasing concentrations of NaCl to the reaction starting from physiological NaCl concentration (150–950 mM). *Prevotella intermedia* is an obligate anaerobic pathogen. Therefore, binding experiments with *P. intermedia* 25611 were also conducted under anaerobic conditions in order to rule out whether the presence of oxygen during the binding reaction leads to increased FI acquisition. At least three independent experiments were performed in duplicates; averages and standard deviations (SD) were calculated for evaluation of the data.

### Binding of FI from serum by *P. intermedia* strains

Maxisorp microtitre plates (Nunc, Roskilde, Denmark) were coated over night at 4°C with 10 µg/ml (67 nM) of the polyclonal rabbit anti human FI antibody PK9205 in coating buffer (15 mM Na_2_CO_3_, 35 mM NaHCO_3_, pH 9.6). *P. intermedia* strains as well as *S. aureus* ATCC 25923 and Newman (FI binding positive control) and *E. coli* DH5α (FI binding negative control) were taken from agar plates and resuspended in gelatin-veronal buffer (GVB) (144 mM NaCl, 1.8 mM Na-barbital, 3.1 mM barbituric acid, 0.1% gelatin, pH 7.35) to obtain an OD_600_ of 0.5 and harvested by centrifugation thereafter. The pellet was resuspended in 1/10 volume GVB and bacteria prepared in duplicates for the FI consumption assay. Human serum, which had been incubated at 56°C for 30 min (heat inactivated normal human serum, HI-NHS), 50 µl diluted to 2% in GVB was mixed with 50 µl of the bacterial suspension and samples were incubated for 1 h at RT and subsequently spun down. Thereafter, 50 µl of the supernatants were added to the wells of the microtiter plate, which was washed with immunowash (50 mM Tris-HCl, 150 mM NaCl, 0.1% (v/v) Tween 20, pH 7.5), blocked with quench solution (immunowash with 0.3% fish gelatin, Nordic, Täby, Sweden) for 1 h at 37°C and washed again before adding the supernatants. Then, plates were incubated for 1 h at RT. Thereafter, plates were washed three times with immunowash followed by addition of the primary antibody (goat anti-human FI A313 diluted 1∶2,500 in quench solution). The plates were incubated for 1 h at RT and washed thrice as indicated above. Then, the secondary antibody (HRP-conjugated rabbit anti-goat, dilution 1∶2,500) was added to the wells and the plates were incubated again for 1 h at RT. After three washing steps plates were developed using 1,2-phenylenediamine dihydrochloride (OPD tablets, Dako, Glostrup, Danmark) and read at 490 nm in a Cary50 Bio UV spectrometer, 50MPR microplate reader (Varian, Palo Alto, CA). Values measured for wells containing only the bacteria without HI-NHS where the primary antibody had been omitted were used for background normalization. The relative amount of FI bound to *P. intermedia* was calculated as follows: The value from wells with 1% HI-NHS in GVB without bacteria was set to 100%. The amount by which total FI in the supernatants was reduced was considered to be bound by bacteria and the relative amount depleted by bacteria was expressed as percent FI bound from 1% HI-NHS. Averages of duplicates were used for calculations; data sets of three independent experiments were analyzed by calculating averages and SD.

### Activity of proteins bound to *P. intermedia* ATCC 25611

*P. intermedia* ATCC 25611 was picked from plates, resuspended in PBS, 1% BSA pH 7.0 to obtain an OD_600_ of 0.5 and harvested by centrifugation. Resulting pellets were finally resuspended in 1/100 volume of PBS, 1% BSA, pH 7.0. *P. intermedia* is known to produce proteases such as Interpain A, able to degrade C3 and C4 alpha chains [37]. Therefore, in order to minimize background degradation by *P. intermedia* ATCC 25611 aliquots (20 µl, approximately 2×10^9^ bacteria) of the bacterial suspensions were incubated for 30 min at 56°C besides adding 2 mM E-64 (Sigma-Aldrich, St. Louis, MO) together with the substrates C3 or C4 later when preparing samples for the degradation reaction. The protein binding assays were performed in a total reaction volume of 40 µl. Bacteria were either mixed with 5 µg, 10 µg and 15 µg FI (final concentration 125 ng/µl (1.42 µM), 250 ng/µl (2.84 µM) and 375 ng/µl (4.26 µM)) or C4BP (final concentration 125 ng/µl (0.22 µM), 250 ng/µl (0.44 µM) and 375 ng/µl (0.66 µM)), or 1 µg, 2 µg and 5 µg FH (final concentration 0.025 µg/µl (0.16 µM), 0.05 µg/µl (0.32 µM), 0.125 µg/µl (0.8 µM)) and further incubated for 1 h at RT. Finally, bacteria were washed once with 50 mM Tris, 150 mM NaCl, pH 7.4 and transferred to fresh reaction tubes.

To determine whether FI bound to *P. intermedia* ATCC 25611 retained its protease activity, degradation assays with ^125^I-C4b were performed. Bacteria were mixed with 2 mM E-64 to prevent unspecific degradation by cysteine proteases. Thereafter, C4met (50 µg/ml; 0.26 µM), C4BP (100 µg/ml; 0.18 µM), trace amounts of ^125^I-C4b and 50 mM Tris, 150 mM NaCl, pH 7.4 were added in a final reaction volume of 50 µl. Samples were incubated for 4 h at 37°C, centrifuged and resulting supernatants were run on a 10–15% SDS-PAGE gradient gel. Gels were dried and protein bands visualized by autoradiography using a radioisotope imager (Fujifilm, Tokyo, Japan). Bacteria, which were preincubated with buffer without FI served as a negative control and reference for quantification of the results. Further controls were samples in which bacteria were omitted and either FI (5.28 µg/ml; 60 nM) and C4BP (100 µg/ml; 0.18 µM) (positive control) or only FI (5.28 µg/ml; 60 nM) without cofactor (negative control) were added. Data were quantified with the Image Gauge Software (Fujifilm, Tokyo, Japan).

To assess cofactor activity of bound C4BP in degradation assays, the effect of surface-bound C4BP on C4b degradation was investigated. In these experiments, FI (5.28 µg/ml; 60 nM) was added instead of C4BP after the binding reactions with C4BP were completed. The negative control without bacteria as described above contained C4BP (100 µg/ml; 0.18 µM) instead of FI. Finally, all samples were incubated for 1.5 h at 37°C.

Functional integrity of FH bound to *P. intermedia* ATCC 25611 was assessed in degradation assays with ^125^I-C3b. Briefly, bacterial suspensions were prepared as indicated above, mixed with 2 mM E-64, C3met (150 µg/ml; 0.81 µM), FI (5.28 µg/ml; 60 nM), and trace amounts of ^125^I-C3b. The final volume was adjusted to 50 µl by adding 50 mM Tris, 150 mM NaCl, pH 7.4. Samples were incubated for 1.5 h at 37°C and thereafter treated as described above. In addition to the negative control, in which FH was omitted, an additional negative control without bacteria was included, in which FI was omitted. Moreover, a positive control without bacteria containing FH (20 µg/ml; 32 µM) and FI (5.28 µg/ml; 60 nM) was prepared.

Results obtained from the degradation assays were evaluated using one-way ANOVA followed by Tukey's post-hoc test on samples containing bacteria and compared to bacteria-containing samples, which had been incubated with buffer instead of FI, C4BP, or FH.

In order to rule out false positive binding by bacteria treated with elevated temperature and to show that binding of the respective proteins occurs under these conditions, *P. intermedia* ATCC 25611 was incubated at 56°C for 30 min and binding assays were performed as described with the ^125^I-labeled proteins; binding of FI, C4BP and FH was compared to binding of these proteins by untreated bacteria.

The contrast and brightness levels of images showing gels of degradation assays were minimally adjusted in whole images using Photoshop software (Adobe Systems, San Jose, CA) on a Macintosh computer (Apple, Cupertino, CA). Relevant lanes were selected and pictures were trimmed using the same software.

### Statistical analysis

One-way ANOVA followed by Tukey's Multiple Comparison Test was used for statistical evaluation of the data applying GraphPad Prism software (La Jolla, CA).

## Results

### *P. intermedia* strains bind purified FI and FI in complete serum

**Figure 1:**
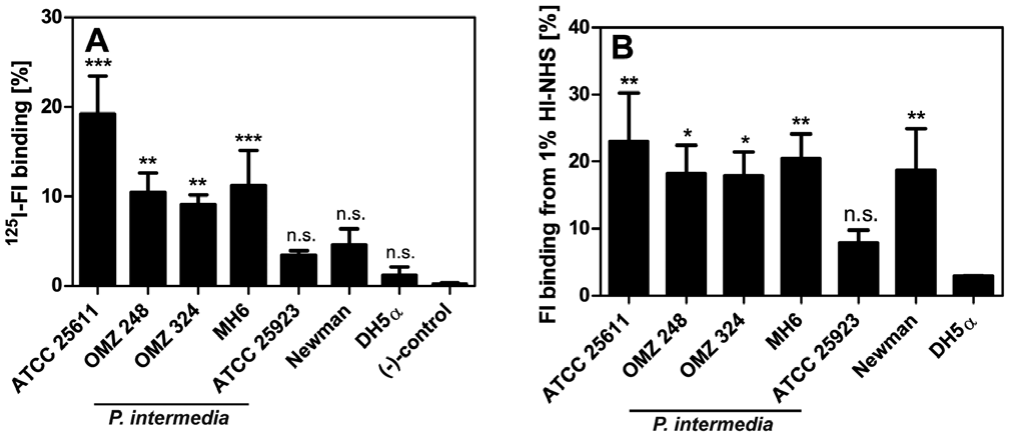
*P. intermedia* strains bind purified ^125^I-FI and FI from HI-NHS. A) *P. intermedia* ATCC 25611, OMZ 248, OMZ 324, MH6, *S. aureus* strains ATCC 25923 and Newman (FI binding positive control) as well as *E. coli* DH5α (FI binding negative control) were incubated with ^125^I-FI (250 kcpm). Bound and free FI were separated by centrifugation through sucrose, the radioactivity associated with pellets and supernatants was measured in a gamma counter and the percentage of radioactivity bound to the pellet was calculated. Samples containing ^125^I-labeled FI that was incubated with buffer alone served as a negative control. Averages of three independent experiments performed in duplicates are shown; error bars show SD. One-way ANOVA followed by Tukey's post-hoc test was used for statistical evaluation of the data as compared to the negative control (*** p<0.001; ** p<0.01, n.s., not significant). B) HI-NHS (1%) was mixed with *P. intermedia* ATCC 25611, OMZ 248, OMZ 324, MH6 or *S. aureus* strains ATCC 25923 and Newman, as well as *E. coli* DH5α and samples were incubated for 1 h at RT followed by centrifugation. Aliquots of the supernatants were added to the wells of a microtiter plate coated with the polyclonal anti-FI antibody PK9205. Captured FI was detected with a polyclonal goat anti-FI antibody. Binding of FI to bacteria resulted in a decreased FI concentration in the serum. This value was then used to calculate bound FI. The graph shows averages of three independent experiments performed in duplicates and error bars indicate SD. One-way ANOVA followed by Tukey's post-hoc test was applied for statistical evaluation of the data as compared to values calculated for *E. coli* DH5α (** p<0.01; * p<0.05, n.s., not significant).

We chose the type strain *P. intermedia* ATCC 25611 as well as three clinical isolates to investigate FI acquisition by these Gram-negative pathogens. Besides the Gram-positive *S. aureus* strains ATCC 25923 and Newman (internal positive controls) we included also a Gram-negative bacterium *E. coli* DH5α, as a negative control. *P. intermedia* ATCC 25611, OMZ 248, OMZ 324, MH6 bound purified FI whereas no significant binding was observed for *E. coli* DH5α as well as *S. aureus* ATCC 25923 and Newman using this experimental setup (Fig. 1A). The binding capacity of both *S. aureus* strains was remarkably low, however consistent with the reported binding properties of this species. Binding of FI by *S. aureus* has been found to be sensitive to the presence BSA [38], which was a buffer supplement in our binding experiments. In order to evaluate the role of BSA on FI binding we performed an experiment where we omitted BSA and found that BSA had only a minimal effect on FI binding (data not shown). FI binding appeared to be a slightly elevated in the absence of BSA, however the effect was not statistically significant. Other factors than BSA, such as culture conditions, might affect FI binding by *S. aureus* [39] and result in our inability to detect significant levels of FI binding by previously reported binders *S. aureus* ATCC 25923 and Newman.

Next we sought to prove that *P. intermedia* strains selectively bind FI from serum. To this end, we used HI-NHS to avoid risk of detecting FI bound to C4b/C3b deposited on the bacterial surface. Thus, we investigated the consumption of FI from 1% HI-NHS in the presence of the various *P. intermedia* strains as well as *E. coli* DH5α (FI binding negative control) and *S. aureus* ATCC 25923 and Newman (FI binding positive controls). This experimental set up was chosen in order to avoid potential artifacts caused by direct binding of antibodies to *P. intermedia* [40], [41]. All our *P. intermedia* strains acquired significant amounts of FI from HI-NHS as compared to FI consumption by *E. coli* DH5α (Fig. 1B). *S. aureus* Newman bound FI from serum significantly in contrast to the results obtained from binding experiments with purified ^125^I-FI. *S. aureus* ATCC 25923 did not consume significant amounts of FI from HI-NHS as compared to *E. coli* DH5α. These data confirm that *P. intermedia* strains also acquire FI under more physiological conditions in the presence of all serum proteins.

**Figure 2:**
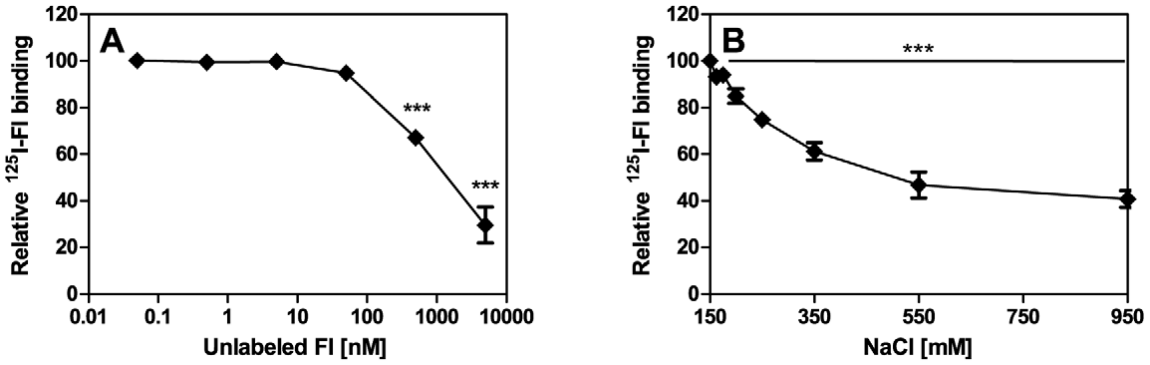
Competition of ^125^I-FI binding by *P. intermedia* with unlabelled FI and sensitivity of FI binding to ionic strength. A) Bacteria were mixed with unlabelled FI (final concentrations 0–5000 nM/0–0.44 µg/µl) and incubated with 250 kcpm ^125^I-FI for 1 h at RT and bound protein was measured as described in Fig. 1. B) Increasing concentrations of NaCl (150–950 mM) were added together with 250 kcpm ^125^I-FI to bacteria. Averages of three independent experiments performed in duplicates are shown with error bars indicating SD and FI acquisition expressed as protein bound relative to protein bound without addition of competitor. One-way ANOVA followed by Tukey's post-hoc test was used for statistical analysis (*** p<0.001).

The specificity of FI binding by *P. intermedia* was confirmed by reducing significantly binding of ^125^I-FI by strain ATCC 25611 with unlabeled FI (Fig. 2A). The interaction between FI and its bacterial ligand was intermediately sensitive to increasing ionic strength as the binding in the presence of 550 mM NaCl was decreased only by 50% thus indicating that the binding is only partially determined by ionic interactions (Fig. 2B). The presence of oxygen during the binding reaction did not lead to false positive binding, for example, due to new epitopes accessible upon oxygen exposure as similar results were obtained when experiments were performed in anaerobic conditions (data not shown).

### FI bound to *P. intermedia* ATCC 25611 retains its serine protease activity

**Figure 3:**
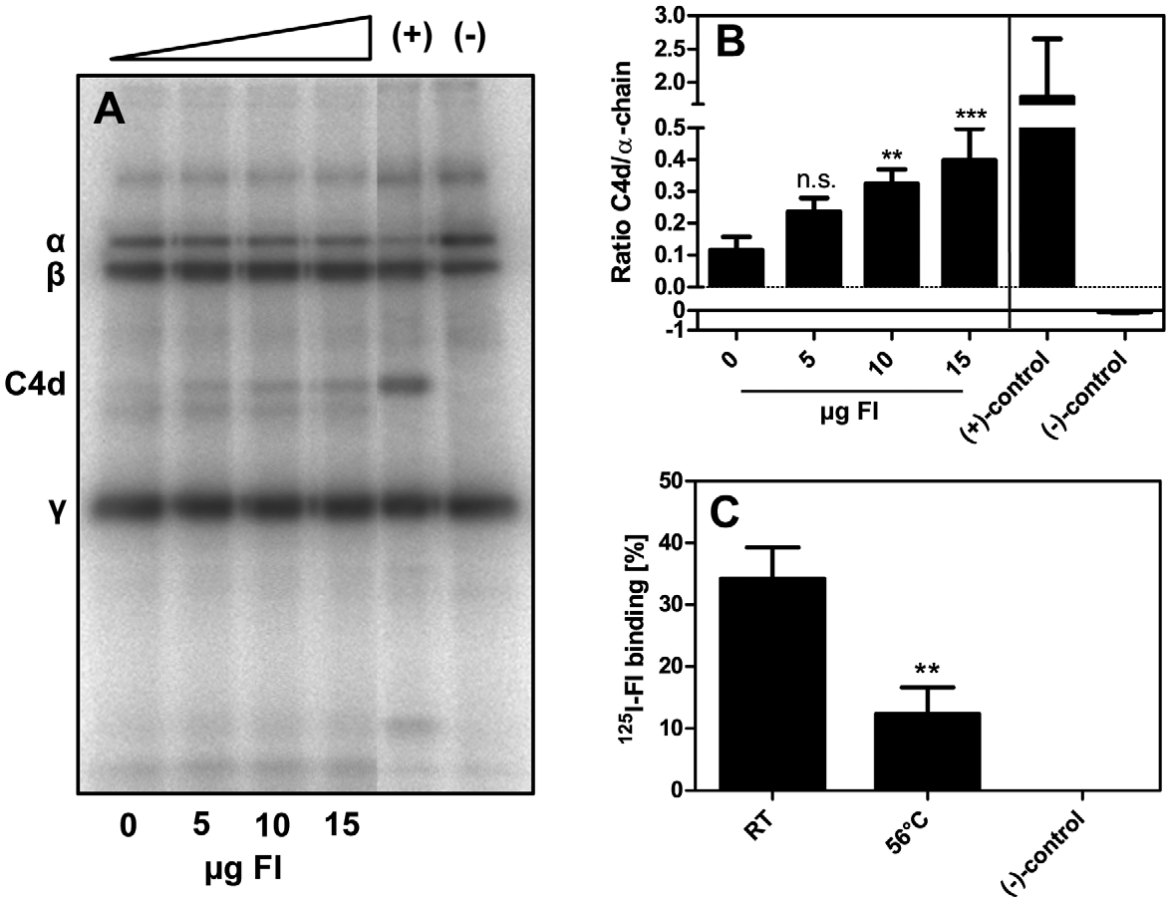
Degradation of ^125^I-C4b by FI bound to *P. intermedia*. A) *P. intermedia* ATCC 25611 was incubated at 56°C for 30 min and subsequently 5 µg, 10 µg, and 15 µg FI (final concentration 125 ng/µl (1.42 µM), 250 ng/µl (2.84 µM) and 375 ng/µl (4.26 µM)) were added to the bacterial suspension and samples incubated for 1 h at RT. Thereafter, bacteria were washed and mixed with E-64, C4met, C4BP and ^125^I-C4b. Samples were incubated for 4 h at 37°C, centrifuged to remove bacteria followed by separation of proteins on a 10–15% SDS-PAGE gradient gel under reducing conditions. Gels were dried and ^125^I-C4b was detected using a phosphorimager. Controls were samples only containing purified proteins without bacteria; either FI and C4BP (positive control) or only FI without cofactor (negative control) were added to degradation reactions. B) Data were quantified using the Image Gauge Software. Averages of four independent experiments are shown, error bars denoting the SD. The ratio of C4d/α-chain was calculated and data from samples containing FI bound to bacteria compared to the control containing bacteria without FI using one-way ANOVA followed by Tukey's post-hoc test applied to samples containing bacteria (*** p<0.001; ** p<0.01; n.s., not significant). C) Effect of elevated temperature on FI binding by *P. intermedia*. *P. intermedia* ATCC 25611 was incubated at 56°C or RT for 30 min and a binding assay was performed with 250 kcpm ^125^I-labeled FI to exclude false positive binding by bacteria treated at elevated temperature. Data were compared to values measured for samples incubated at RT using one-way ANOVA followed by Tukey's post-hoc test (** p<0.01).

The data above indicate that *P. intermedia* strains bind FI possibly providing protection from activated complement on the level of C3 convertase. The retention of protease activity of FI is an obligatory prerequisite for such a protective role of FI. To test whether FI bound to *P. intermedia* retains its serine protease activity, bacteria were first incubated at 56°C for 30 min in order to minimize background degradation by bacterial proteases and then incubated with purified FI. This procedure was adopted from the routinely applied procedure to prepare serum for immunological and protein binding experiments. Thereafter, FI activity was tested with ^125^I-labeled C4b and C4BP as a cofactor. Cleavage of C4b after incubation of the bacteria with increasing amounts of FI resulted in an augmentation of the signal intensity of the C4d band (Fig. 3A and B). The presence of bacteria alone caused some background degradation resulting predominantly in a product migrating faster than C4d. In order to exclude false positive binding of FI due to the exposition of bacteria to elevated temperature, binding of ^125^I-FI to *P. intermedia* ATCC 25611 was tested after 30 min incubation of bacteria at RT or 56°C. We observed that heat treatment caused a decrease in FI binding, which might be due to partial denaturation of the bacterial ligand (Fig. 3C). Thus this treatment does not cause an artifactual increase in FI binding. Overall, these results indicate that FI bound to *P. intermedia* ATCC 25611 retains its protease activity resulting in specific degradation of C4b.

### *P. intermedia* strains bind FI cofactors C4BP and FH

**Figure 4:**
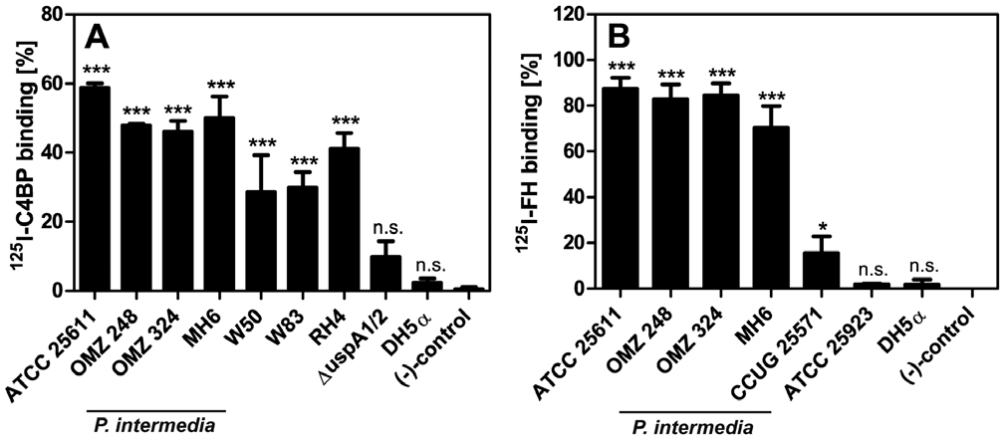
*P. intermedia* isolates bind purified FI cofactors C4BP and FH. The indicated *P. intermedia* strains, as well as A) *M. catarrhalis* RH 4, *P. gingivalis* W50 and W83 (C4BP binding positive controls), *M. catarrhalis* Δ*uspA*1/2, and *E. coli* DH5α (C4BP binding negative controls) or B) *S. pyogenes* CCUG 25571 (FH binding positive control), *S. aureus* ATCC 25923, and *E. coli* DH5α (FH binding negative controls) were mixed with (A) 500 kcpm ^125^I-C4BP or (B) ^125^I-FH and incubated for 1 h at RT. Proteins bound to bacteria were detected as described in Fig. 1. Samples containing ^125^I-labeled proteins that were incubated with buffer alone served as negative controls. Bars represent averages of three independent experiments performed in duplicates and SD are indicated as error bars. One-way ANOVA and Tukey's post-hoc test was used for statistical analysis as compared to negative controls (*** p<0.001; * p<0.05; n.s., not significant).

Since *P. intermedia* strains bind FI we tested whether these strains also bind FI cofactors C4BP and FH. All *P. intermedia* strains significantly bound C4BP (Fig. 4A) that was comparable to *M. catarrhalis* RH4 and *P. ginigivalis* W83 and W50 (C4BP binding positive controls). *M. catarrhalis* has been shown previously to acquire C4BP on its surface via the ubiquitous surface proteins UspA1 and UspA2 [24] and we found that the *uspA*1/*uspA*2 deletion mutant of RH4 (C4BP binding negative control) did not bind C4BP that was consistent with published data. Likewise, *E. coli* DH5α (C4BP binding negative control) did not acquire significant amounts of C4BP. In parallel to the C4BP-binding, all our *P. intermedia* strains also bound FH (Fig. 4B). *S. pyogenes* CCUG 25571 (FH binding positive control) has previously been shown to bind FH [30], however binding appeared to be less efficient than acquisition of FH by *P. intermedia* strains in this experimental set-up, whereas *S. aureus* ATCC 25923 as well as *E. coli* DH5α (FH binding negative controls) did not bind FH.

**Figure 5:**
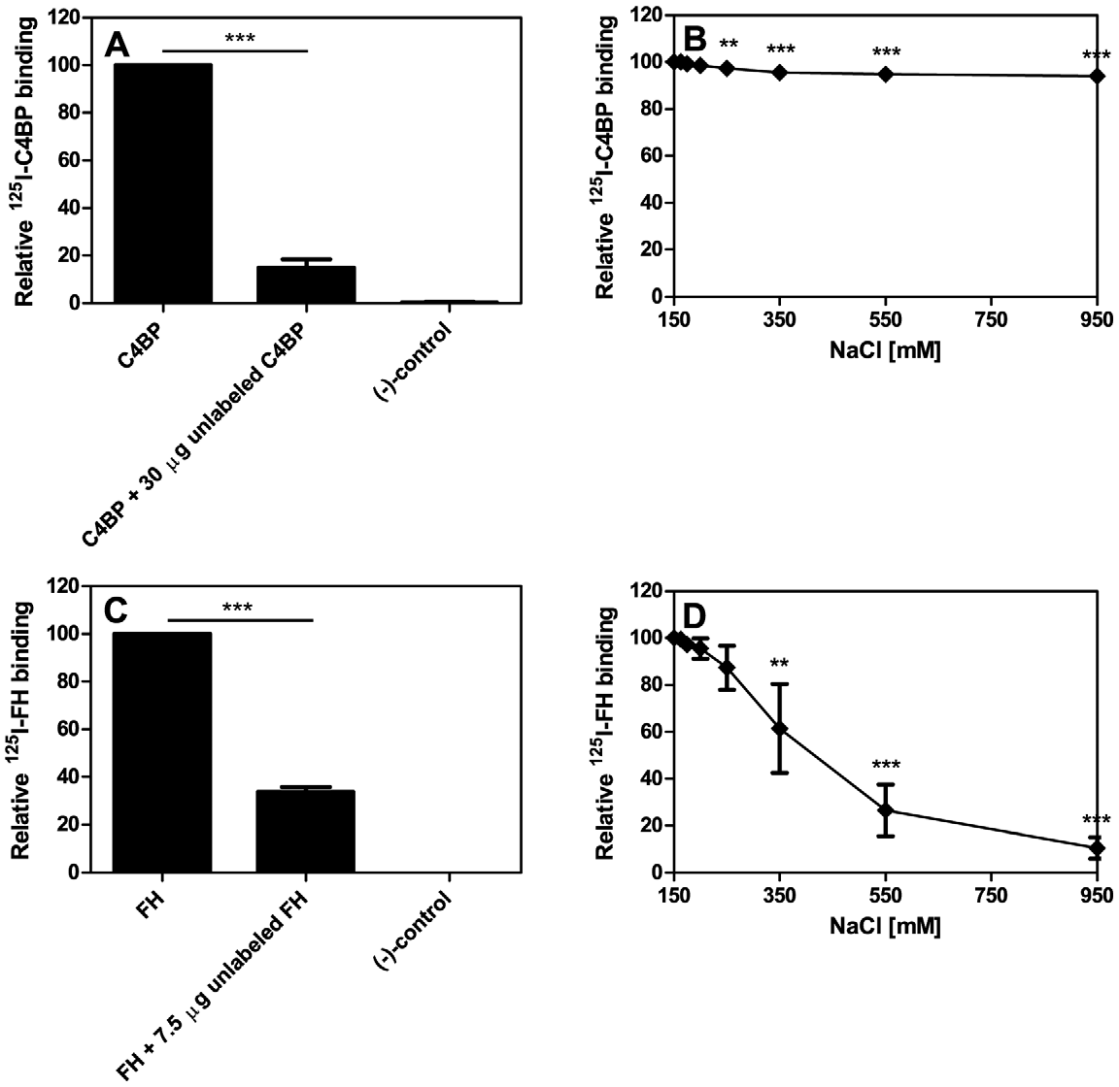
Specificity and sensitivity of C4BP and FH binding by *P. intermedia* ATCC 25611. A) Binding of 250 kcpm ^125^I-C4BP was competed by using 30 µg (final concentration 1.32 µM) unlabeled C4BP, C) ^125^I-FH binding by *P. intermedia* ATCC 25611 was competed by 7.5 µg (final concentration 1.25 µM) unlabeled FH. The decrease of ^125^I-C4BP and ^125^I-FH binding was evaluated comparing the results of samples with and without radiolabeled protein and bacteria. Samples without bacteria served as negative controls. The sensitivity of the interaction to ionic strength was tested adding increasing concentrations of NaCl to binding reactions with B) ^125^I-C4BP and D) ^125^I-FH. Three independent experiments were performed in duplicates; averages and SD were calculated and binding expressed as protein bound relative to protein bound without addition of competitor. One-way ANOVA followed by Tukey's post-hoc test was applied for statistical evaluation of the data (** p<0.01; *** p<0.001).

The binding of ^125^I-C4BP bound to *P. intermedia* ATCC 25611 could be reduced significantly by adding unlabelled C4BP (Fig. 5A) and increasing ionic strength affected the interaction between ^125^I-C4BP and bacteria only negligibly (Fig. 5B) implying hydrophobic interaction. Further competition experiments with FH revealed that ^125^I-FH could also be competed by adding unlabelled FH (Fig. 5C). The interaction of *P. intermedia* ATCC 25611 with FH was abrogated with increasing concentrations of NaCl (Fig. 5D) suggesting an ionic binding mode for FH.

### Binding of C4BP and FH by *P. intermedia* ATCC 25611 leads to increased degradation of C4b and C3b, respectively

**Figure 6:**
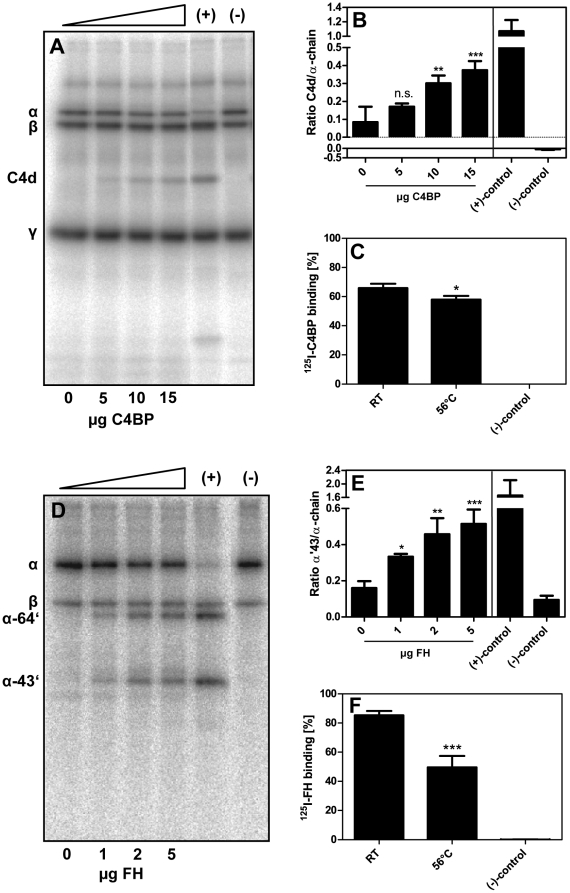
C4BP and FH bound to *P. intermedia* ATCC 25611 retain their cofactor activity. *P. intermedia* ATCC 25611 was incubated for 30 min at 56°C and subsequently incubated with C4BP (5–15 µg) (final concentration 125 ng/µl (0.22 µM), 250 ng/µl (0.44 µM) and 375 ng/µl (0.66 µM)) (A) and B)) or FH (1–5 µg) (final concentration 0.025 µg/µl (0.16 µM), 0.05 µg/µl (0.32 µM), 0.125 µg/µl (0.8 µM)) (D) and E)) for 1 h at RT. Samples without C4BP and FH were used as a reference. Bacteria were washed and transferred to fresh reaction tubes. A) and B) Bacteria were mixed with E-64, C4met, FI and trace amounts of ^125^I-C4b. Samples were incubated for 1.5 h at 37°C, centrifuged and proteins separated on a 10–15% SDS-PAGE gradient gel under reducing conditions. Samples without bacteria, which contained C4BP but no FI served as negative controls. Samples supplemented with C4BP and FI were positive controls in this experimental setup. B) and E) Protein bands were quantified using the Image Gauge Software. C) and F) *P. intermedia* ATCC 25611 was incubated at 56°C for 30 min and binding assays were performed as described earlier with ^125^I-labeled protein. D) and E) Bacterial suspensions were mixed with E-64, C3met, FI and trace amounts of ^125^I-C3b. Samples were incubated for 1.5 h at 37°C and separated as described above. A negative control without bacteria was included, in which FI was omitted. A positive control without bacteria supplemented with FH and FI was prepared. All experiments were performed in triplicates, the mean values and SD were calculated. One-way ANOVA and Tukey's post-hoc test were used for statistical analysis on bacteria containing samples. Degradation mediated by cofactors bound to *P. intermedia* was evaluated by comparing the ratios of C4d or α′43 degradation products to α-chains of samples where protein was omitted during preincubation (*** p≤0.001; ** p<0.01; * p<0.05; n.s., not significant).

To test whether the binding of FI cofactors led to increased complement inactivation by degradation of C4b and C3b we preincubated heat-treated *P. intermedia* ATCC 25611 with increasing amounts of C4BP and upon further incubation in the presence of FI we found augmented generation of C4d (Fig. 6A). Quantification of the signals confirmed a statistically significant increase in degradation of C4b in the presence of bound C4BP (Fig. 6B). Bacterial cells incubated at elevated temperature bound ^125^I-C4BP equally well as those remaining at RT (Fig. 6C).

FH bound to *P. intermedia* ATCC 25611 also retained its function as a cofactor for FI mediated cleavage of C3b. Thus we found concentration dependent increase in band intensity of the 64 kDa and 43 kDa degradation products of C3b (Fig. 6D) quantified by densitometry (Fig. 6E). Heat treatment of *P. intermedia* ATCC 25611 resulted in a decrease of ^125^I-FH binding to bacteria (Fig. 6F).

Taken together, the results presented here show that cofactors C4BP and FH required for proteolytic activity of FI retain their cofactor activity when bound to *P. intermedia* ATCC 25611.

## Discussion

The ability to acquire complement inhibitors is an important virulence mechanism and provides various bacterial pathogens with a major advantage. To date, with a sole exception, only the binding of the cofactors of FI by bacterial pathogens has been reported. FI acquisition has exclusively been associated with the Gram-positive *S. aureus* so far. Initially, it had been shown that C3b inactivation on the surface of *S. aureus* is mediated by FI and FH and in this first study binding of these proteins has been described [38]. Cleavage of C3b was independent of the presence of cofactors [38] and led to decreased phagocytosis efficiency by human polymorphonuclear cells [22]. Clumping factor A could be identified as the ligand for FI binding and a shed fragment of clumping factor A served as a cofactor for FI-mediated cleavage of C3b [20], [21].

Our results suggest that FI in addition to cofactor acquisition might be an important virulence mechanism of *P. intermedia*, the infection of which is proposed to be of local, disseminated, acute, as well as chronic nature [2]–[6] implying versatile solutions of this pathogen to cope with the host response in the environment of various niches, including gingival crevicular fluid in gingival or periodontal pockets or in the lungs of patients afflicted with aspiration pneumonia [42]. Previously, it was shown that the periodontal pathogens *P. intermedia* and also *P. gingivalis* produce proteases, which degrade complement proteins. Interpain A of *P. intermedia* degrades the α-chain of purified C3 and this proteolytic inactivation of C3 reduces the bactericidal activity of NHS and makes interpain A expressing strains more resistant to killing by complement [37]. A similar effect on NHS was also observed for *P. gingivalis* strains W50 and W83. The proteases HRgpA, RgpB and Kgp (gingipains) interfere with complement activation with HRgpA and RgpB being most effective [43]. The mechanism underlying this observation is predominantly the degradation of C3, C4 and C5 α-chains [43], [44]. Interestingly, interpain A and gingipains exhibit a synergistic inhibitory activity on complement activation [37]. This is in consistency with a model proposed for periodontal biofilms in which both pathogens cooperate to prepare a milieu favoring biofilm formation with help of their secreted proteases. The bacterial proteases of *P. intermedia* and *P. gingivalis* cause biphasic effects on complement activation, at low concentrations leading to activation of complement by C1 deposition and generation of anaphylatoxins C3a and C5a. Low degree of inflammation may be beneficial for the pathogens at this stage of infection. Upon establishment of *P. intermedia* and *P. gingivalis* at the site of infection, higher concentrations of proteases lead to complement inhibition by degradation of C3, C4 and C5 [45], [46]. Binding of FI, C4BP and FH might be a crucial mechanism of complement evasion at low protease levels. Accumulation of complement inhibitors of the host on bacteria might even represent the backbone of *P. intermedia* immune evasion, presumably being a rather passive interaction with structural components of the bacterial envelope than being dependent on secretion of active proteases. Furthermore, disseminating bacteria are not protected by the environment of the biofilm in the periodontal pocket. Thus, acquisition of complement inhibitors in the blood stream might provide the bacteria with the ability to expand disease to a systemic level.

The production of the aforementioned proteases by periodontal pathogens including *P. intermedia* made it necessary to distinguish between inherent proteolytic activity of the bacteria themselves and degradation mediated by FI and cofactors acquired by bacteria. To this end it was necessary to adjust assay conditions by using protease inhibitors and gentle heat treatment, which was identical to that routinely used for inactivation of serum. Incubation of the bacteria at 56°C caused a decrease in protein binding, which was negligible in case of C4BP but still significant for all the proteins examined in this study and could be due to partial denaturing of the bacterial ligands potentially occurring during incubation at this temperature. However, we sought to prove that no artifactual binding of proteins upon heat treatment led to false positive results testing the biological activity of protein occupied by its ligand and we found no increase in protein acquisition. Therefore, we conclude that FI, C4BP or FH bound to *P. intermedia* ATCC 25611 after incubation at 56°C for 30 min retain their activity in degrading C3b or C4b. The binding studies with these proteins show a specific and strong binding to all *P. intermedia* strains tested and reduced binding capacity of bacteria after heat treatment does not affect the activity of the proteins bound by bacteria. It appears worth noting that we observed high binding for the three complement inhibitors presented here in this study. However, it is not a general phenomenon of *P. intermedia* strains to bind a broad variety of complement proteins in an unspecific manner. Besides FI, C4BP, and FH we also tested binding of the membrane cofactor protein CD46 in a similar experimental set-up and observed very low binding (below 5%) for the *P. intermedia* strains tested (data not shown).

Taken together, besides complement evasion mediated by proteases produced by *P. intermedia*, complement inhibitor acquisition might be beneficial at low concentrations of the inherent proteases, and various mechanisms have to be regarded as pieces of the puzzle drawing the complete picture of immune evasion, preparation of an ideal microenvironment, and potentially dissemination of *P. intermedia*.

Moreover, binding of FH to bacterial cells promotes adherence and invasion of epithelial cells as has been shown for *Streptococcus pneumoniae*. Glycosaminoglycans on host cells serve as a receptor for FH mediated adherence of *S. pneumoniae* subsequently followed by integrin dependent internalization of the bacilli [47]. Similar to FH, C4BP interacts with ligands on host cells thereby also possibly promoting bacteria host cell interaction. Heparin and chondroitin sulfate are glycosaminoglycan ligands known to be involved in C4BP and FH host cell interaction [48]–[50]. If adherence to host cells is mediated by C4BP and FH bound by *P. intermedia* will be subject of future studies.

Here we provide data indicating that FI, C4BP and FH acquisition might be an additional mechanism of complement evasion. Binding of these complement inhibitors draws the attention towards host proteins, in particular complement, bound by *P. intermedia* and its significance during the course of infection. Detailed investigation of the role of complement inhibitor acquisition in interaction with host cells and definition of the bacterial ligands of this interplay will be addressed in the future in order to contribute to the understanding of the role of *P. intermedia* in outcome of disease.
